# Effects of Maternal Caffeine Exposure During Gestation on Neurobehavior, Glucose Metabolism and Circadian Rhythm in Offspring Rats

**DOI:** 10.3390/toxics14100893

**Published:** 2026-10-07

**Authors:** Minying Sun, Huanxin Pan, Guangyu Yang, Yan Zhang, Juntao Li, Xudong Li, Wei Zhu

**Affiliations:** 1 Guangzhou Center for Disease Control and Prevention (Guangzhou Health Supervision Institute), Guangzhou 510440, China; gzcdc_sunmy@gz.gov.cn (M.S.); gzcdc_yanggy@gz.gov.cn (G.Y.); gzcdc_zhangy@gz.gov.cn (Y.Z.); gzcdc_lijt@gz.gov.cn (J.L.); lxdnutrition@163.com (X.L.); 2 Institute of Public Health, Guangzhou Medical University & Guangzhou Center for Disease Control and Prevention (Guangzhou Health Supervision Institute), Guangzhou 510440, China; 3 Guangzhou Panyu District Center for Disease Control and Prevention, Guangzhou 511400, China; panhx5@126.com

**Keywords:** pregnancy, caffeine, neurobehavior, glucose metabolism, circadian rhythm

## Abstract

Caffeine intake can have numerous effects on neurological and cognitive impairments. However, its influences on offspring still need to be evaluated. This study aimed to investigate the effects of caffeine exposure during gestation on behavioral development, metabolic function, and circadian rhythms in offspring. A total of 96 Sprague–Dawley rats were included, with an equal number of males and females. After confirming pregnancy, the female rats were randomly divided into four groups on average and administered caffeine at three distinct doses (5, 20, and 80 mg/kg/day) or equivalent volumes of normal saline throughout the gestational period. The health status and weight gain of pregnant rats, as well as the litter size and body weight of offspring, were continuously monitored. Open field test, Morris water maze, intraperitoneal glucose tolerance tests (IPGTT) and intraperitoneal insulin tolerance tests (IPITTs) were performed. The expression levels of 7 circadian clock genes (*Bmal1*, *Clock*, *Cry1*, *Cry2*, *Per1*, *Per2*, and *Rev-erbα*) were quantified. This revealed a significant reduction in maternal weight gain in the 80 mg/kg caffeine group compared to controls. Although the birth weight of offspring was not significantly affected, the postnatal weight gain of offspring was slower in caffeine groups. Behavioral assessments demonstrated elevated spontaneous activity and increased anxiety-like behaviors in caffeine-exposed offspring. Metabolic evaluations indicated impaired glucose tolerance specifically in female offspring, though no significant alterations were observed in insulin sensitivity, spatial learning and memory performance. Maternal caffeine exposure during gestation also disrupted the rhythmicity of hepatic circadian clock gene expression in offspring rats. These findings suggest that gestational caffeine exposure exerts selective adverse effects on offspring development, behavior, glucose metabolism and circadian rhythm. Caffeine consumption during pregnancy necessitates careful consideration.

## 1. Introduction

Caffeine, a xanthine derivative primarily sourced from coffee, tea, and soft drinks, is also found in chocolate, cocoa-containing foods, pharmaceutical preparations, over-the-counter medicines, and some dietary supplements [1]. The liver serves as the primary organ for caffeine metabolism, yielding three key metabolites: paraxanthine, theobromine, and theophylline [2]. Factors influencing caffeine metabolism include genetics, age, gender, physical condition, smoking, alcohol consumption, as well as specific foods and medications (e.g., grapefruit, broccoli, oral contraceptives, rifampicin) [3]. During pregnancy, the activity of caffeine-metabolizing enzymes is reduced, leading to a prolonged half-life of caffeine. This results in easier transmission of caffeine and its metabolites across the placental barrier and through breast milk. According to the Developmental Origins of Health and Disease (DOHaD) concept, adverse environmental exposures during early life, such as the fetal and infant periods, may exert persistent effects on long-term health through epigenetic regulation and metabolic programming, thereby increasing the risk of chronic diseases and mental illnesses later in life [4,5]. Therefore, caffeine exposure during gestation may have potential toxic effects such as adverse pregnancy outcomes (fetal growth restriction or low birth weight), impaired brain development (neurobehavioral abnormalities), and metabolic dysfunction (insulin resistance, diabetes and hyperlipidemia) in offspring [6,7,8]. Caffeine is teratogenic in animal studies when administered at high concentrations, but current epidemiologic evidence is conflicting and not adequate to confirm the risk of congenital anomalies resulting from maternal caffeine consumption [9,10,11].

The circadian rhythm refers to the intrinsic 24 h cycle established by an organism to synchronize with external environmental cues, encompassing processes such as sleep–wake patterns, body temperature fluctuations, heart rate variability, physical activity, and hormone secretion [12]. The generation and maintenance of circadian rhythm are orchestrated by the rhythmic expression of clock genes, including *Per1*, *Per2*, *Per3*, *Cry1*, *Cry2*, *Clock*, *Bmal1*, Casein kinase I epsilon (*CKIε*), and *Rev-erbα* [13,14]. In mammals, the intracellular clock mechanism operates through interplaying positive and negative transcriptional feedback loops that govern the cyclic fluctuations in RNA and protein levels of pivotal clock components [15]. During early fetal development, the circadian rhythm of the fetus is entrained by maternal rhythmic signals. Studies have demonstrated that caffeine has the potential to perturb circadian rhythms by modulating the expression of circadian clock genes and influencing melatonin secretion [16,17]. This raises the question of whether maternal caffeine exposure during pregnancy could exert lasting effects on the circadian rhythm of offspring.

Animal experiments and cohort studies have found that perinatal caffeine exposure can influence birth weight and early neurodevelopment of offspring. It also has long-term effects on liver function, glucose and lipid metabolism; the endocrine system; reproductive system; behavior; and cardiovascular and cerebrovascular function. In particular, some of these effects show gender differences [18,19,20]. The similarity in metabolism between humans and rodents makes it useful for exploring the effects and molecular mechanisms of prenatal caffeine exposure on offspring. However, existing studies often focused on the effects of a certain aspect of function or used a single dose of caffeine exposure, which was generally considered a “safe” dose. The results of different studies are not consistent. In this study, we aimed to investigate the effects of maternal caffeine exposure during gestation on neurobehavior, glucose metabolism and circadian rhythm in offspring rats. Furthermore, three distinct doses (5, 20, and 80 mg/kg/day) of caffeine were used to validate whether there was a dose–response relationship.

## 2. Materials and Methods

### 2.1. Animals

A total of 96 Sprague–Dawley rats (male:female = 1:1), aged 7–8 weeks, were obtained from Hunan SJA Laboratory Animal Co., Ltd. (Changsha, China). The animals were housed in a 12 h:12 h light/dark cycle with lights on at 7:00 a.m. and lights off at 19:00 p.m. Zeitgeber time 0 (ZT0) was defined as the time of lights on (7:00), and ZT12 was defined as the time of lights off (19:00). The environmental conditions were strictly controlled (temperature 22 ± 2 °C and humidity 50–70%). Food and water were adequately supplied and freely consumed. After acclimatization for a week, female rats were paired with males until pregnancy was confirmed by the presence of vaginal plugs and sperm in vaginal smears. The day of confirmation was designated as gestational day 0 (GD0). Pregnant rats were randomly assigned to either the control group or caffeine exposure groups.

All experiments, including animal care and sample collection protocols, were performed in accordance with the ARRIVE guidelines and were approved by the Animal Ethics Committee of the Guangzhou Center for Disease Control and Prevention. Animals were maintained under standard laboratory conditions following the Guide for the Care and Use of Laboratory Animals and the internationally accepted 3R (Replacement, Reduction, Refinement) principles for humane experimental animal use. This study was conducted in full compliance with the ARRIVE guidelines (Animal Research: Reporting of In Vivo Experiments) and adhered to the Animals (Scientific Procedures) Act 1986 (UK), EU Directive 2010/63/EU on the protection of animals used for scientific purposes, and the NIH Guide for the Care and Use of Laboratory Animals (National Research Council, Washington, DC, USA).

### 2.2. Treatment

**Figure 1:**
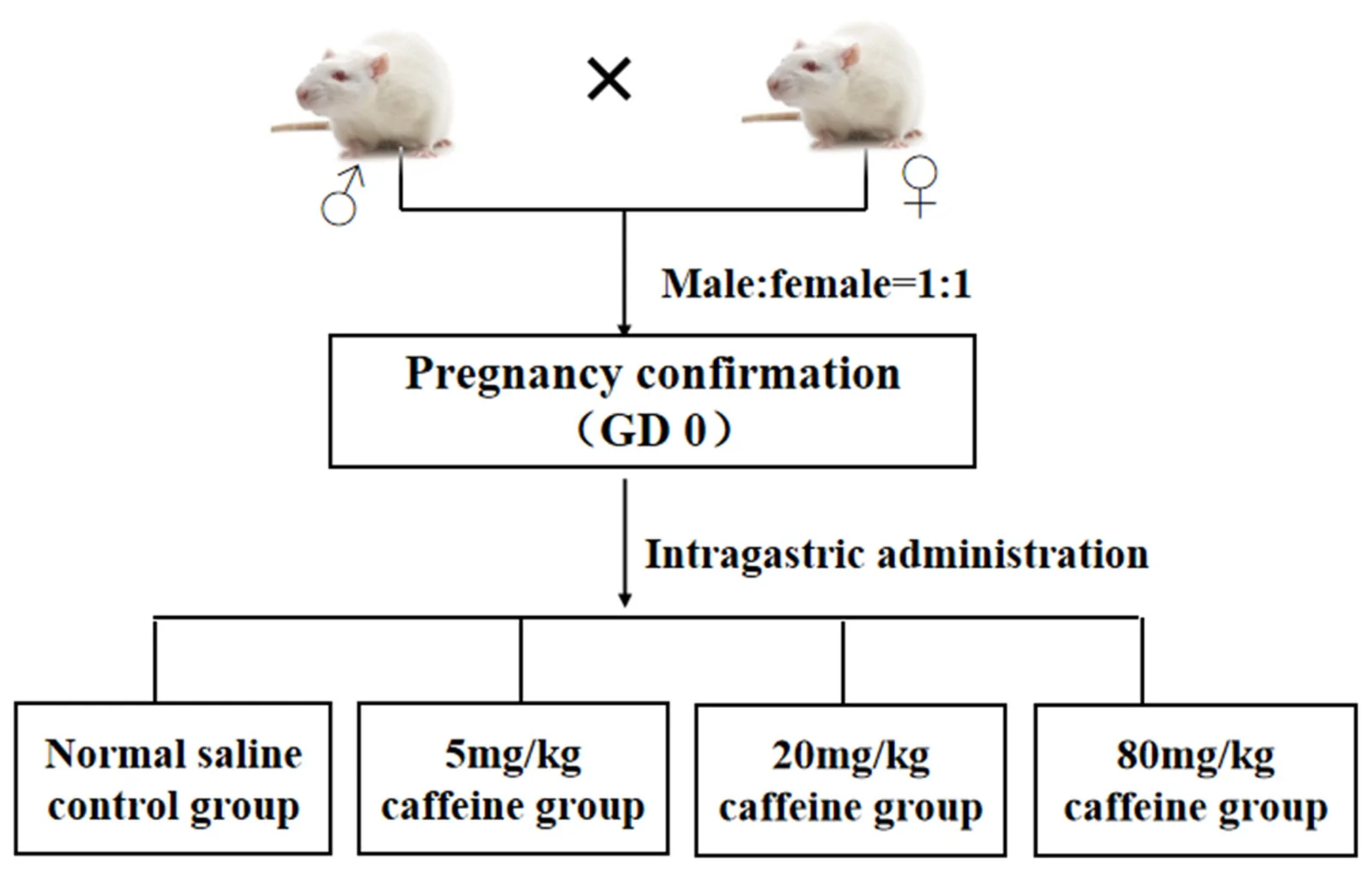
The flow chart of the animal experiment intervention.

American College of Obstetricians and Gynecologists (ACOG) and the European Food Safety Authority (EFSA) recommend that pregnant women should consume no more than 200 mg of caffeine per day [21,22]. Based on the maximum daily caffeine intake of 200 mg for a 60 kg pregnant woman, the medium dose of 20 mg/kg for rats was calculated by the body surface area (BSA) normalization method. According to preliminary experiments, the low-dose and high-dose groups were set at 5 mg/kg and 80 mg/kg. During the entire pregnancy period, approximately 21–23 days, pregnant rats in the caffeine exposure groups were administered caffeine (Sigma-Aldrich, St. Louis, MO, USA, purity > 99%) via gavage at doses of 5, 20, and 80 mg/kg b.w. daily at the same fixed time (08:00–09:00 a.m.), while the control group received an equivalent volume of normal saline. The pregnant rats were individually housed in cages until delivery, with monitoring of general activity levels, food and water intake, weight gain, and overall health status every day. The day of birth was designated as postnatal day 0 (PD0). Only fetal rats from litters containing 8–18 pups were included, and there was consistency in the number of offspring among different groups. The number of pups was adjusted to 8 (♂:♀ = 1:1) per litter within 24 h to ensure sufficient and equal nutrition during lactation. Following birth, the weight of offspring rats was continuously monitored. After weaning, male and female rats were housed separately. The flow chart of the animal experiment intervention was shown in Figure 1.

### 2.3. Open Field Test

At postnatal week 7 (PW7), offspring rats were subjected to an open-field test to evaluate both locomotor activity and anxiety-related behaviors. The open-field apparatus was constructed from black plastic plates measuring 75 cm × 75 cm × 46 cm. Each rat was placed in the center of the arena, and its spontaneous movements were recorded using a video camera (open field test analysis system, Flyde, Guangzhou, China) positioned above the area for a duration of 10 min. Following each session, the open-field apparatus was cleaned with 95% ethanol to eliminate any residual animal odors that could potentially impact subsequent tests. Animal behavior analysis software was utilized to calculate parameters such as total distance traveled, central distance, peripheral distance, and activity time within the open field.

### 2.4. Morris Water Maze

The Morris water maze (MWM) is utilized to assess the spatial learning ability and memory of offspring rats (PW11). The MWM apparatus comprises a cylindrical pool (160 cm in diameter and 45 cm in depth), a video camera system, and video analysis software (Morris water maze video analysis system, Jiliang, Shanghai, China). The pool is partitioned into four quadrants with the center of the bottom of the pool serving as the central point, and black spatial markers of various shapes are affixed around the wall of each quadrant. A circular platform with a diameter of 9 cm and a height of 30 cm is randomly positioned in the center of a quadrant, and water is added to the pool until the water level is 2 cm above the platform, with the water temperature maintained at 24 ± 2 °C. A camera is mounted on a stand 210 cm above the pool and linked to video analysis software on a computer to monitor the movements of the rats. Each rat completes four trials per day to locate a submerged platform within a maximum swim time of 90 s. The escape latency for swimming to the platform is indicative of short-term memory. Following the completion of the trials, the platform is removed, and the animals are assessed for long-term memory 24 h later. The percentage of time spent in the target quadrant (the quadrant previously containing the platform) and the number of target approaches are quantified.

### 2.5. IPGTT and IPITT

At PW14, a glucose tolerance test was conducted following a 16 h overnight fast, while an insulin tolerance test was performed 5 h after food removal. Glucose (Sigma-Aldrich, USA; 2 g/kg body weight) and insulin (Novo Nordisk, Copenhagen, Denmark; 0.75 U/kg body weight) were administered intraperitoneally. Blood samples were collected from the tail tip at 0, 15, 30, 60, 90, and 120 min post glucose or insulin injection. Then blood glucose was measured immediately using a portable glucometer with matching test strips (Accu-Chek Performa, Roche Holding AG, Basel, Switzerland).

### 2.6. Blood and Tissue Collection

At PW16, the offspring rats were euthanized at ZT1 (08:00), ZT6 (13:00), ZT11 (18:00), ZT16 (23:00) and ZT21 (04:00) by intraperitoneal administration of 2.5% pentobarbital sodium anesthesia. Experiments conducted during the dark phase were carried out in a dim red-light environment (<5 lx). All rats underwent a 24 h fasting period prior to euthanasia to minimize the impact of food consumption. Abdominal aortic blood samples were collected and centrifuged at 3000 rpm for 10 min. The resulting plasma was stored at −80 °C until the day of detection for glucose, cholesterol, and triglycerides using an automatic biochemical analyzer (Drew Scientific Inc., Miami Lakes, FL, USA). The livers were excised, weighed, flash-frozen in liquid nitrogen for a brief period, and then stored at −80 °C until further analyses.

### 2.7. Quantitative RT-PCR

Hepatic total RNA was isolated using TRIzol reagent (Life Technologies, Carlsbad, CA, USA). First-strand cDNA was synthesized from approximately 500 ng of RNA using the PrimeScript TM RT Master Mix (Takara Bio Inc., Kusatsu, Shiga Prefecture, Japan) following the manufacturer’s protocol. Real-time PCR was conducted with the SYBR Premix Ex Taq TM II kit (Takara Bio Inc., Kusatsu, Shiga Prefecture, Japan). The expression levels of 7 circadian clock genes (*Bmal1*, *Clock*, *Cry1*, *Cry2*, *Per1*, *Per2*, and *Rev-erbα*) were quantified using the Applied Biosystems Quant Studio TM 7 Real-Time PCR system (Thermo Fisher, San Diego, CA, USA) in duplicate. β-actin served as the internal control. The relative gene expression was determined using the 2^−ΔΔCt^ method, and the daily expression patterns were presented as fold changes relative to the mean level of the control group at ZT0.

### 2.8. Statistical Analysis

All data were expressed as means ± standard deviation (SD). Maternal weight, postnatal weight of offspring, average escape latency, and glucose levels in the IPGTT/IPITT were analyzed using two-way repeated measures analysis of variance (ANOVA) with Dunnett’s post hoc test. Pregnancy outcomes, data from the open field test, and the area under the curve (AUC) of IPGTT/IPITT were analyzed using one-way ANOVA or non-parametric tests, with Dunnett’s post hoc test for pairwise comparisons. Metabolites, hormones, and clock gene expression levels across all groups were compared using two-factor (time×treatment) ANOVA, with Dunnett’s post hoc tests. AUC was calculated using GraphPad Prism 5.0 (GraphPad Software, Inc., San Diego, CA, USA). R (version 4.4.1, R Foundation for Statistical Computing, Vienna, Austria) was utilized, with significance set at *p* < 0.05. Figures were generated using GraphPad Prism 5.0. Cosinor analysis was conducted to assess diurnal rhythmicity (SigmaPlot 14.0; Systat Software Inc., San Jose, CA, USA).

## 3. Results

### 3.1. Maternal Weight Gain, Pregnancy Outcomes, and Postnatal Weight of Offspring

**Figure 2:**
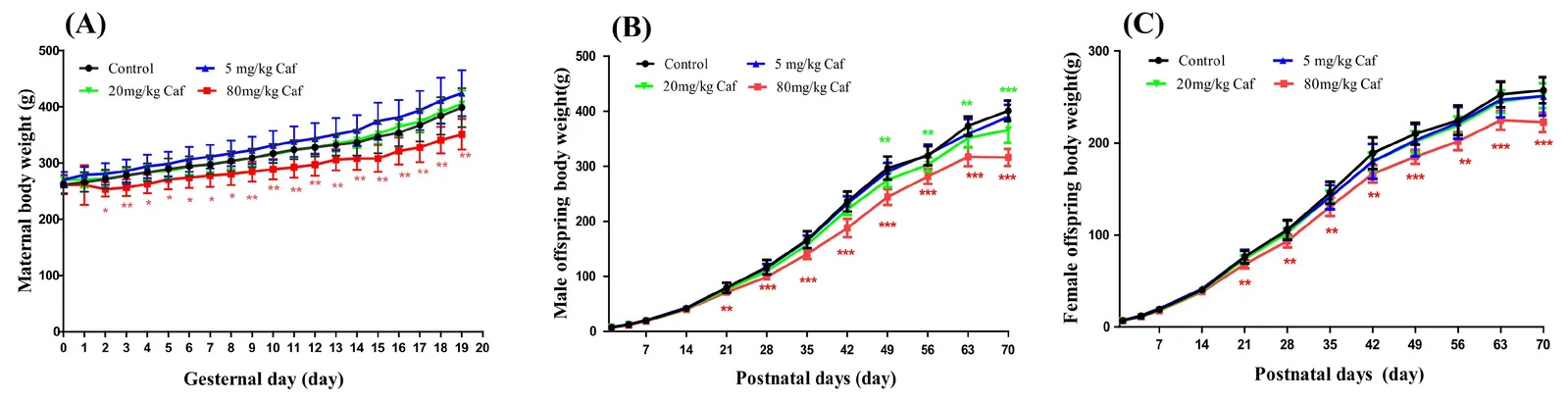
Maternal weight and postnatal weight of offspring. The maternal weight (**A**) and postnatal weight of offspring (**B**,**C**) were continuously monitored. The study included 10–12 pregnant rats or 12 offspring rats per group. All data are presented as Mean ± SD. Statistical analysis was conducted using two-way repeated measures ANOVA with Dunnett’s post hoc test. Significant differences are denoted as follows: *: *p* < 0.05, **: *p* < 0.01, ***: *p* < 0.001 compared to the control group.

During pregnancy, all dams appeared healthy with no signs of distress, abnormal behavior, or reduced mobility. Caffeine exposure significantly reduced maternal weight gain in the 80 mg/kg caffeine group compared to the control group (*p* = 0.006; Figure 2A). However, no significant differences were observed in the other groups. There were no significant differences in gestation length, litter size, and pups’ birth weight between the caffeine exposure groups and the control group (Supplementary Table S1). Following birth, the weight of offspring rats was continuously monitored. The body weight of male offspring was reduced in the 20 and 80 mg/kg caffeine groups compared to the control group (*p* = 0.011; *p* < 0.001, respectively; Figure 2B). The 80 mg/kg caffeine group exhibited a significant decrease in weight gain of female offspring compared to the control group (*p* < 0.001; Figure 2C). No significant differences were found in the other groups.

### 3.2. Open Field Test

**Figure 3:**
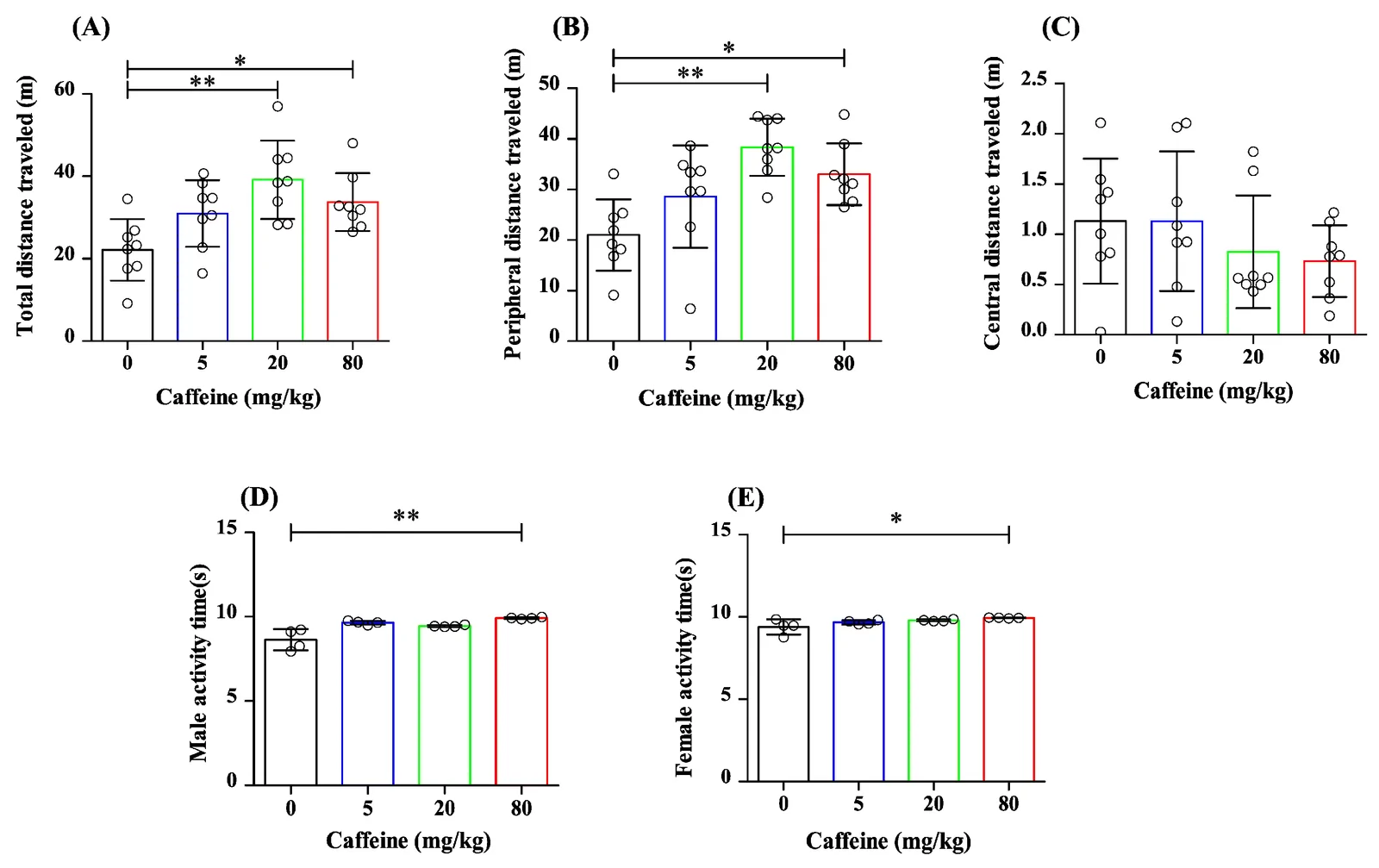
Evaluation of locomotor activity and anxiety in offspring rats. Total distance traveled (**A**), peripheral distance traveled (**B**), central distance traveled (**C**), and activity time (**D**,**E**) in the open field apparatus were recorded and calculated with an open field test analysis system. N = 8 offspring rats (male:female = 1:1) per group. Data are presented as Mean ± SD, with individual data points shown as scatter dots. Statistically significant differences were detected by one-way ANOVA with Dunnett’s post hoc test (**A**–**C**) and by non-parametric test with Dunnett’s post hoc test (**D**,**E**). *: *p* < 0.05, **: *p* < 0.01 vs. the control group.

There were no gender differences in the total distance, central distance, and peripheral distance traveled by the offspring in the open field. Therefore, male and female data were combined for analysis. The total distance traveled in the open field significantly increased in the 20 and 80 mg/kg caffeine groups compared to the control group (*p* = 0.003; *p* = 0.004, respectively; Figure 3A). Additionally, there were no significant differences observed in the distance traveled in the central zone, but the distance traveled in the peripheral zone increased (*p* = 0.001, *p* = 0.023, respectively; Figure 3B,C). These results suggest that offspring rats exposed to caffeine during pregnancy exhibited increased spontaneous activity and anxiety. Activity time in the open field area also increased for both male and female offspring in the 80 mg/kg caffeine group compared to the control group (*p* = 0.002; *p* = 0.014, respectively; Figure 3D,E).

### 3.3. Morris Water Maze

**Figure 4:**
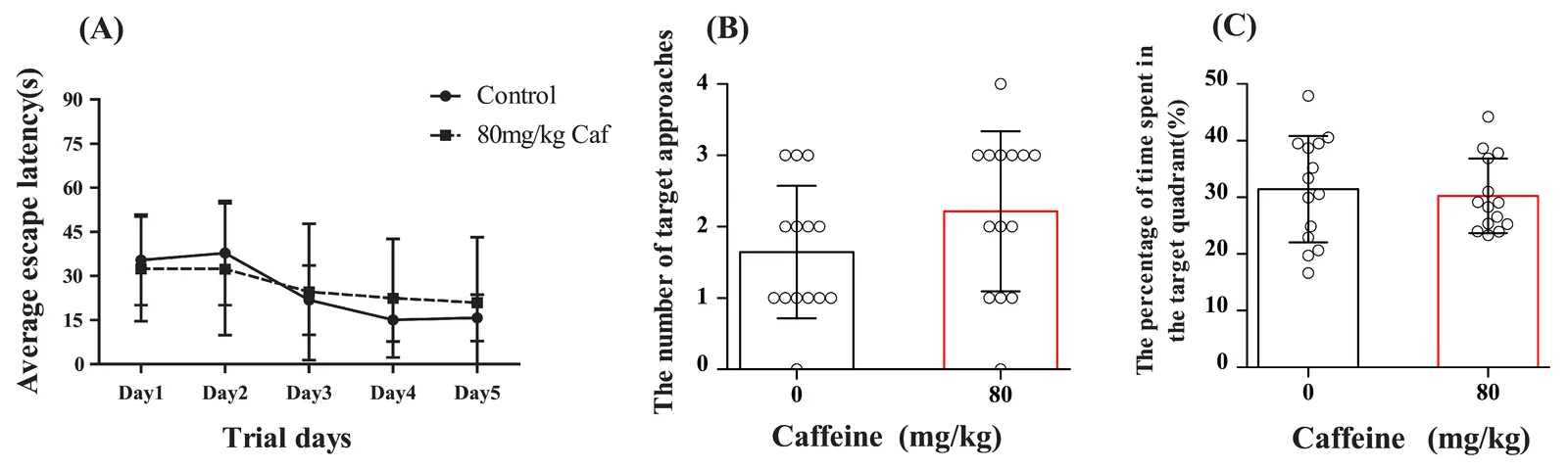
Performance of offspring rats in the MWM task. Average escape latency to find the hidden platform (**A**). The number of target approaches (**B**). The percentage of time spent in the target quadrant (**C**). N = 14 offspring rats (male:female = 1:1) per group. Data are expressed as Mean ± SD, with individual data points shown as scatter dots. Statistically significant differences were detected by two-way repeated measures ANOVA (**A**) and by non-parametric test (**B**,**C**).

No significant sex-based differences in escape latency were observed across the trial days in the MWM task. Therefore, the data obtained from both male and female offspring were pooled for subsequent statistical analyses. Two-way repeated measures ANOVA showed a significant time effect (*p* < 0.001; Figure 4A). However, the average escape latency of the offspring rats did not differ between the control group and the 80 mg/kg caffeine exposure group. After removing the platform, no significant differences were observed in the number of target approaches or the percentage of time spent in the target quadrant between the control group and the 80 mg/kg caffeine exposure group (Figure 4B,C). These results indicate that maternal caffeine exposure did not affect the learning and memory ability of the offspring.

### 3.4. Glucose Tolerance Test

**Figure 5:**
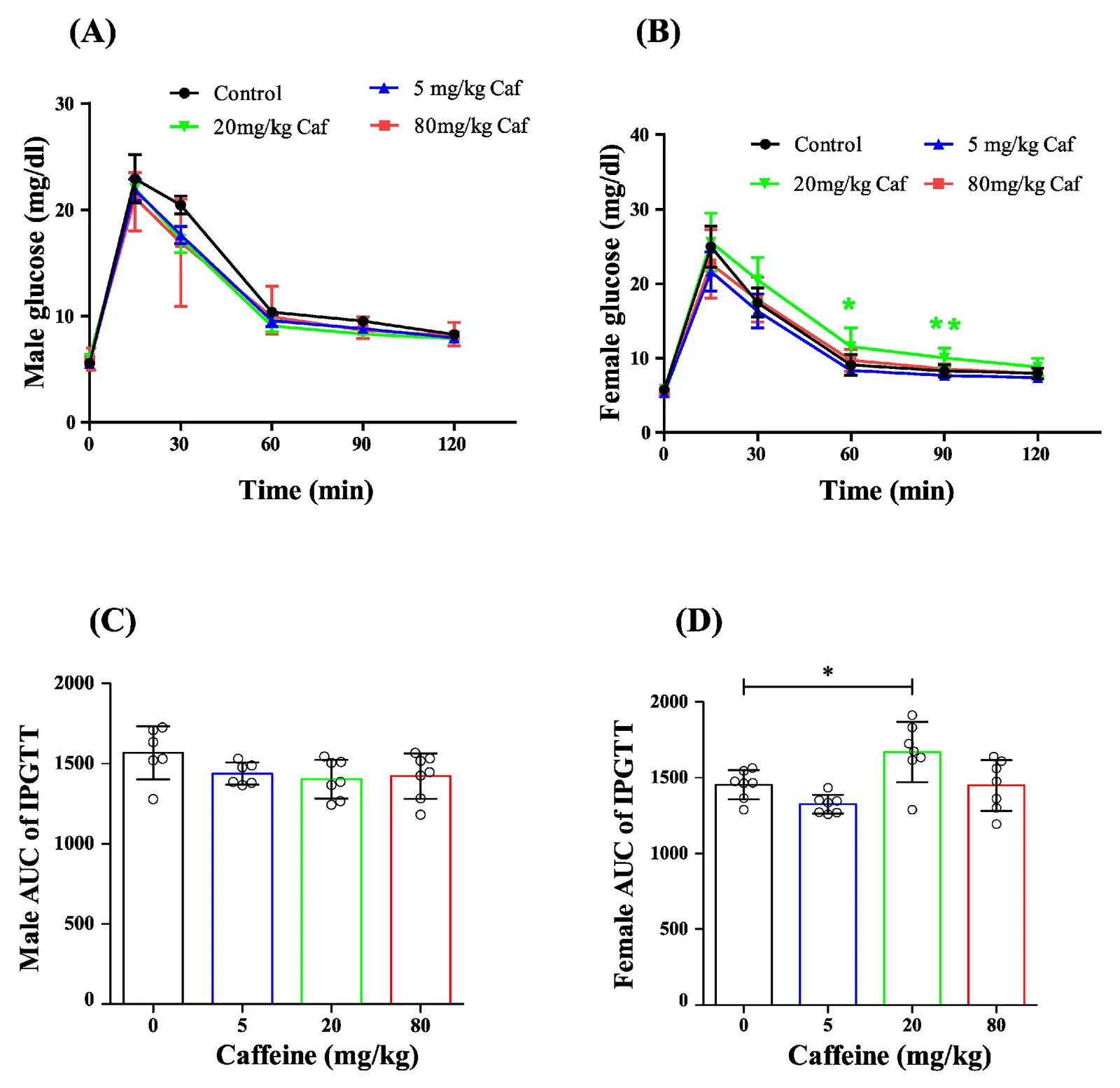
Effects of prenatal caffeine exposure on glucose tolerance in offspring rats. Glucose tolerance tests (**A**,**B**) were performed in offspring rats. The area under the glucose curve for IPGTT (**C**,**D**) was calculated for each rat. The sample size was N = 6–7 male or female offspring rats per time point per group. Data are expressed as Mean ± SD, with individual data points shown as scatter dots. Statistically significant differences were determined using two-way repeated measures ANOVA with Dunnett’s post hoc test and one-way ANOVA with Dunnett’s post hoc test. *: *p* < 0.05, **: *p* < 0.01 vs. the control.

Glucose tolerance data for male and female offspring are presented in Figure 5A and Figure 5B, respectively. The basal levels of blood glucose showed no significant difference between the groups in males or females. One-way ANOVA with Dunnett’s post hoc test revealed that female offspring in the 20 mg/kg caffeine group exhibited significantly higher glucose levels at 60 and 90 min post-injection compared to the control group (*p* = 0.027 and *p* = 0.004, respectively; Figure 5B). The glucose AUC of female offspring in the 20 mg/kg caffeine group was also higher than that of the control group (*p* = 0.024; Figure 5D), indicating impaired glucose tolerance. No significant differences were observed in male offspring. These findings suggest that maternal caffeine exposure during pregnancy may impair the glucose tolerance of female offspring rats.

### 3.5. Insulin Sensitivity

**Figure 6:**
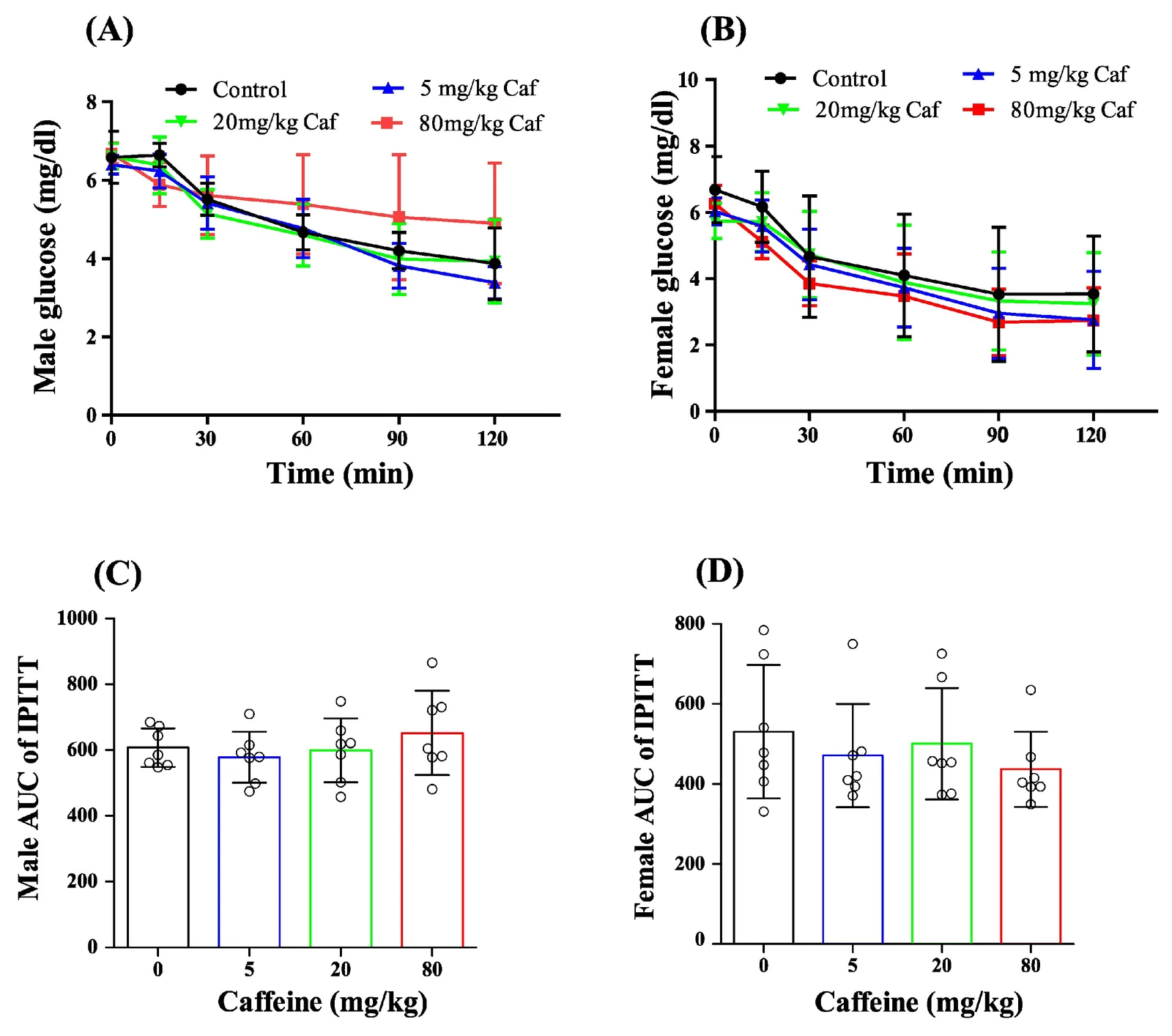
Effects of prenatal caffeine exposure on insulin sensitivity in offspring rats. Insulin tolerance tests (**A**,**B**) were performed in offspring rats. The area under the glucose curve for IPITT (**C**,**D**) was calculated for each rat. The study included 7 male or female offspring rats per time point per group. Data are expressed as Mean ± SD, with individual data points shown as scatter dots. Statistically significant differences were detected using two-way repeated measures ANOVA with Dunnett’s post hoc test (**A**,**B**) and one-way ANOVA with Dunnett’s post hoc test (**C**,**D**).

Insulin tolerance data for male and female offspring are presented in Figure 6A and Figure 6B, respectively. There were no significant differences in baseline blood glucose levels among all the groups in males or females. A two-way repeated measures ANOVA revealed a significant interaction effect in males (*p* = 0.025; Figure 6A). However, no significant differences were observed in insulin-stimulated glucose levels and AUC in both males and females, indicating that maternal caffeine exposure during pregnancy may not impact the insulin sensitivity of offspring rats.

### 3.6. Rhythms of Blood Glucose, Triglycerides, and Total Cholesterol

**Figure 7:**
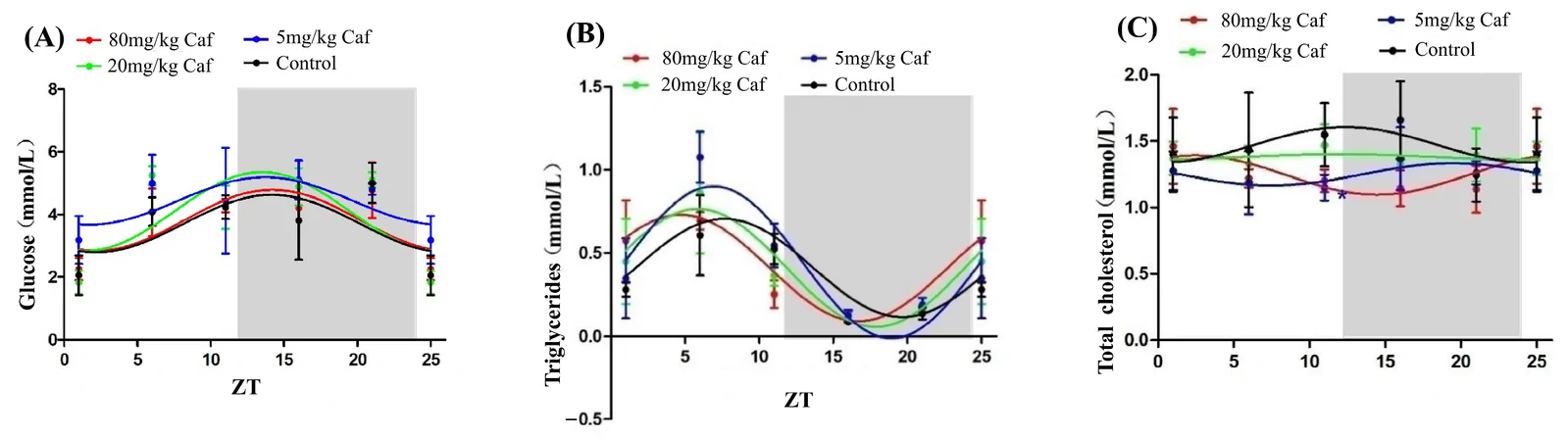
Effects of prenatal caffeine exposure on the rhythms of blood glucose (**A**), triglycerides (**B**), and total cholesterol (**C**). The cosine fitting curves were fitted using the cosine equation y = A + Bcos [2π(x − C)/24] with GraphPad Prism 5.0. All data are expressed as Mean ± SD. *: *p* < 0.05 vs. the control.

At PW10, the changes in plasma glucose, triglycerides, and total cholesterol levels were measured at five time points (ZT1, ZT6, ZT11, ZT16, ZT21) over a 24 h period in male rats (Figure 7). Two-way ANOVA results indicated no statistically significant differences in glucose and triglycerides between the prenatal caffeine exposure group and the control group (*p* = 0.114; *p* = 0.089), while there was a statistically significant difference in total cholesterol levels between the groups (*p* = 0.021). Further pairwise comparisons and one-way ANOVA revealed no differences in blood glucose and triglyceride levels at the five time points, but the total cholesterol level in the low-dose caffeine exposure group showed a statistically significant difference compared to the control group at ZT11 (*p* = 0.036). Cosine analysis results demonstrated that both the prenatal caffeine exposure groups and the control group exhibited strong rhythmic oscillations in blood glucose, triglycerides, and total cholesterol (amplitude test *p* < 0.05). Blood glucose levels showed a diurnal variation with lower levels during the day and higher at night, while triglyceride levels showed an opposite diurnal pattern with higher values during the day and lower at night. However, there were no statistically significant differences in amplitude, peak, and phase compared to the control group.

### 3.7. Liver Clock Gene Expression

**Figure 8:**
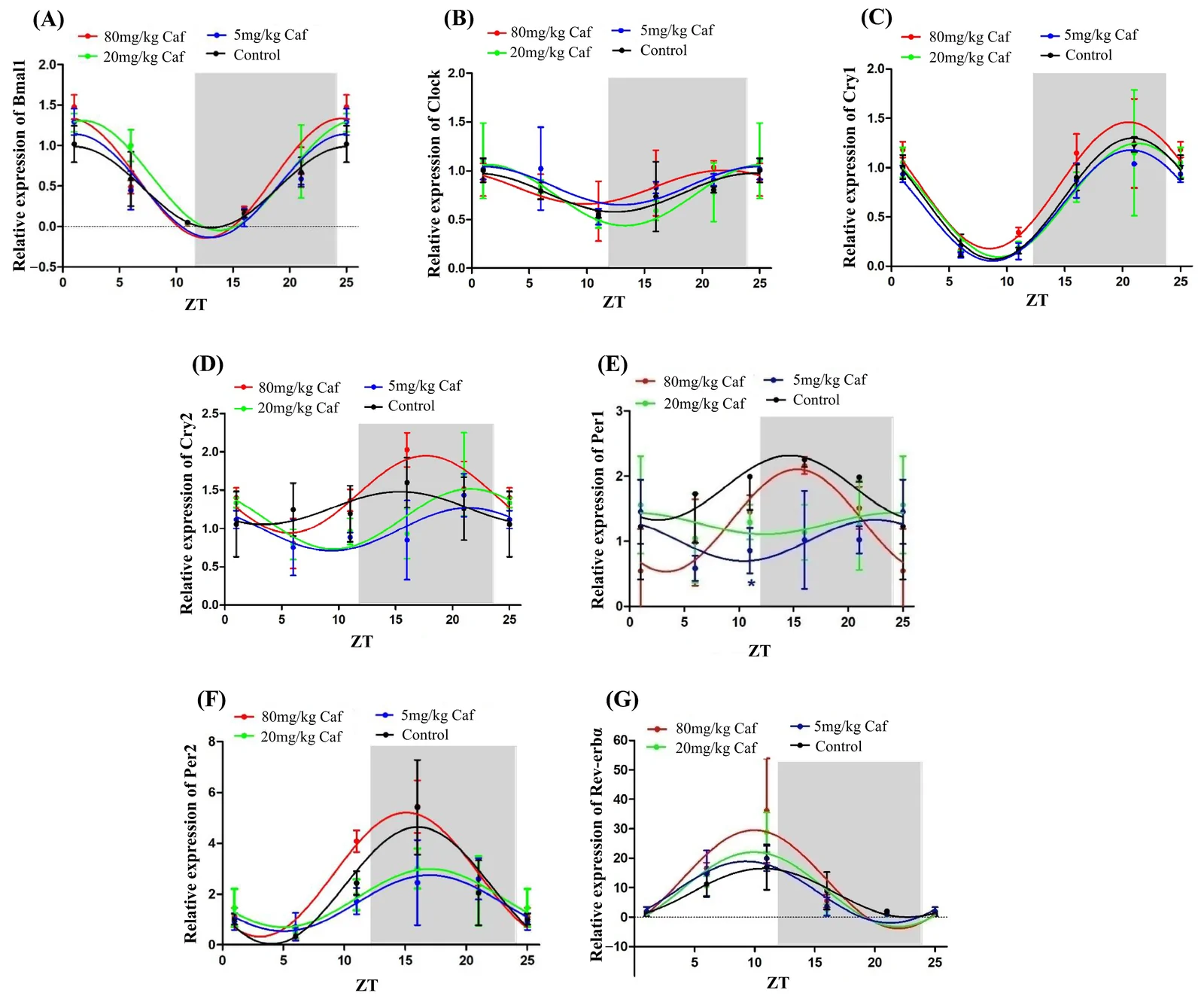
Effects of prenatal caffeine exposure on the expression of circadian clock genes in liver. (**A−G**) showed the expression levels of genes *Bmal1*, *Clock*, *Cry1*, *Cry2*, *Per1*, *Per2*, and *Rev-erbα*, respectively. The cosine fitting curves were fitted using the cosine equation y = A + Bcos [2π(x − C)/24] with GraphPad Prism 5.0. All data are expressed as Mean ± SD. *: *p* < 0.05 vs. the control.

The expression of circadian clock genes in liver mRNA of male rats was also measured at five time points (ZT1, ZT6, ZT11, ZT16, ZT21) over a 24 h period. Two-way ANOVA revealed no statistically significant differences in the gene expression of *Bmal1*, *Clock*, *Cry1*, *Cry2*, *Per2*, and *Rev-erbα* between the exposure groups and the control group, while a significant group difference was observed in the expression of the gene *Per1* (*p* = 0.001). Further pairwise comparisons and one-way ANOVA indicated that the expression of Per1 in the low-dose caffeine exposure group was different from that in the control group at ZT11 (*p* = 0.012). Cosine analysis results demonstrated that both the exposure group and control group exhibited robust rhythmic oscillations in the expression of *Bmal1*, *Clock*, *Cry1*, *Per2*, and *Rev-erbα* (amplitude test *p* < 0.05). However, the amplitude, median, and phase of each exposure group underwent alterations (Figure 8). The expression of *Per1* in low- and medium-dose caffeine exposure groups showed no rhythmicity, and the phase was significantly altered, with a phase delay in the low-dose group and a phase advance in the medium-dose group. The expression of *Cry2* in the control group showed no rhythmicity (*p* > 0.05), but the expression in exposure groups exhibited significant rhythmicity.

## 4. Discussion

The safety and risks of caffeine intake during pregnancy constitute a significant issue in maternal nutrition. A cohort study in the United States revealed that 6.7% of women consumed 101–200 mg of caffeine during pregnancy, and 0.8% exceeded 200 mg per day [23]. A large number of experimental studies have demonstrated that high-dose prenatal caffeine exposure has adverse effects on the metabolism and development of offspring, such as non-alcoholic fatty liver, hypercholesterolemia, diabetes, and growth retardation [24,25,26,27]. However, the evidence regarding caffeine doses generally considered safe remains relatively limited. Our study has investigated the effects of maternal caffeine exposure during gestation on the behavior, glucose metabolism, and circadian rhythm of offspring rats. The experimental groups include three different dose levels: low dose (5 mg/kg), medium dose (20 mg/kg) and high dose (80 mg/kg). This gradient range allows evaluation of both physiologically relevant and excessive gestational caffeine exposure. We aimed to explore the potential impacts of prenatal caffeine exposure in a mammalian model, which may provide insights relevant to human health.

Firstly, in terms of pregnancy outcomes and health status of offspring, studies have indicated that caffeine intake during pregnancy could cause maternal palpitations, insomnia, calcium deficiency, exacerbate morning sickness, and even lead to abnormal uterine contractions resulting in preterm birth and miscarriage. Caffeine can cross the placenta into the fetal bloodstream, increasing the risk of chromosomal abnormalities, reducing fetal oxygen and nutrient supply, leading to low birth weight, abnormal neurodevelopment, and an elevated risk of childhood leukemia [6,28,29]. Our data showed that high-dose caffeine exposure during pregnancy could significantly reduce maternal weight gain and postnatal weight gain in offspring, but had no effect on pregnancy length, litter size, and birth weight. Compared with other studies, the adverse influence of caffeine exposure on maternal and early offspring development observed in this study was relatively mild. Nevertheless, this should not be taken as reassurance that pregnant women may consume caffeinated beverages or foods without concern.

Secondly, in terms of behavioral development, the observed hyperactivity in offspring rats suggested that prenatal caffeine exposure might disrupt neuroendocrine metabolism. Prenatal neurodevelopment exhibits heightened sensitivity to external stimuli, with caffeine, a well-established neuroactive compound, potentially disrupting critical processes such as neural network formation and synaptic connectivity [30,31,32]. These findings align with prior experimental evidence demonstrating behavioral modifications in animal models following gestational caffeine exposure. While the long-term implications of these neurodevelopmental alterations remain incompletely characterized, emerging evidence suggests they may predispose to enduring cognitive impairments and persistent behavioral abnormalities in later life stages [33]. No conclusive evidence was found linking maternal caffeine exposure and learning or memory ability of the offspring. Meanwhile, animal model research has also demonstrated that caffeine modulates hyperoxia-induced decreased neurogenesis in the hippocampus and shows neuroprotective properties in the neonatal rat oxygen toxicity model [34].

Thirdly, in terms of metabolism, prenatal caffeine exposure was detected to significantly influence glucose metabolism in offspring. Our results revealed that offspring rats in the medium-dose caffeine exposure group demonstrated significantly increased blood glucose levels, impaired glucose tolerance, and exhibited gender-specific variations. The underlying mechanisms of this non-linear dose–response relationship may be that moderate doses of caffeine reduce insulin sensitivity through adenosine receptor antagonism, sympathetic activation, and catecholamines, while high doses cause compensatory adenosine receptor upregulation or enhanced hepatic insulin clearance [2,35,36]. In addition, high-dose exposure may indirectly improve/alter glucose homeostasis by reducing food intake or increasing energy expenditure [37]. Our data highlight sex-dependent effects, which have also been shown in other studies that moderate doses (25 mg/kg/day) of caffeine exposure during pregnancy or lactation can cause glucose metabolic and hormonal changes with gender differences [38]. Although no significant differences were observed in IPITT, the absence of plasma insulin measurements limits our ability to distinguish between impaired insulin sensitivity and other contributors to glucose intolerance. These findings carry substantial clinical implications, given the pivotal role of glucose homeostasis in maintaining metabolic equilibrium and preventing the onset of chronic metabolic disorders, particularly diabetes mellitus. Consequently, maternal caffeine consumption or administration may predispose offspring to metabolic dysregulation, suggesting the necessity of caution in caffeine intake during pregnancy [18,39]. Other studies have also shown that indirect exposure through maternal caffeine consumption had the potential to reduce the pathogenesis of type 2 diabetes and improve pancreatic B cell insufficiency in mice [40]. Moreover, a cohort study confirmed that a moderate daily intake of caffeinated beverages during the second trimester could reduce the risk of gestational diabetes by 47% [23].

Furthermore, the circadian rhythm disruptions observed in the offspring rats are particularly intriguing. The circadian clock regulates a wide range of physiological processes, including sleep–wake cycles, hormone secretion, and metabolism [41,42]. Prenatal exposure to caffeine appears to disrupt the normal development of this internal clock, leading to altered sleep patterns, behavioral rhythmicity and many diseases, including metabolic syndrome, obesity, and cancer [43]. The molecular mechanism may be that caffeine exposure regulates circadian clock genes through the glucocorticoid pathway. These genes also directly regulate key enzymes in glucose and lipid metabolism [44,45]. These changes may have far-reaching consequences, impacting not only the daily functioning of the individual but also their long-term health and well-being [46,47]. Perinatal dietary and lifestyle educational policies are needed.

Notably, accumulating evidence from both epidemiological and animal studies challenges the assumption that low-to-moderate caffeine intake during pregnancy is safe. Souza et al. [30] reported that perinatal caffeine intake of 25 mg/kg induced lower maternal body mass and higher plasma cholesterol in pregnant rats. At the same time, it also led to lower birth weight, anxiety-like behavior, and hyperinsulinemia in offspring. Qian et al. [48] emphasized that caffeine disrupted fetal neuroendocrine and metabolic programming even at doses previously considered benign. Our findings extended these observations by demonstrating that low- and medium-dose prenatal caffeine exposure could lead to reduced body weight gain, increased locomotor activity, impaired glucose tolerance, and altered circadian rhythms in offspring. These results added more metabolic and chronobiological consequences to the previously reported disruptions. Furthermore, the impaired glucose tolerance suggested early-life programming of metabolic dysfunction, while the circadian changes might reflect altered development of the suprachiasmatic nucleus or clock gene regulation. Collectively, these data indicated that the dose–response relationship for prenatal caffeine may lack a clear threshold for adverse programming effects. Pregnant women should be advised to limit or avoid caffeine intake, and public health recommendations should be reconsidered.

The mechanisms underlying these effects are likely to be complex and multifaceted. Caffeine is known to interact with multiple molecular targets in the body, including adenosine receptors and calcium channels [20,49]. These interactions may lead to alterations in neural signaling, hormone secretion, and metabolic pathways, ultimately affecting behavior, glucose metabolism, and circadian rhythm [3,50,51]. Animal experimental studies have demonstrated that prenatal caffeine exposure can have different short-term or long-term effects on offspring. Short-term effects mainly involve birth weight, endocrine function, and early neurodevelopmental processes, while long-term effects involve the endocrine system, liver function, glucose and lipid metabolism, cardiac system, reproductive system, and behavior [19,20]. The molecular mechanism may involve changes in signaling pathway activity, gene expression, DNA methylation and histone modification, which provide novel targets to prevent and treat fetal-originated diseases [24,52,53]. However, the specific molecular and cellular pathways involved in these effects still require further studies to fully verify and elucidate.

It is essential to acknowledge that, although the current study offers valuable insights into the impacts of prenatal caffeine exposure, it is subject to certain limitations. The utilization of rats as experimental models came with inherent constraints, and the findings might not be readily extrapolated to humans. In addition, the study concentrated on three doses and durations of caffeine exposure, leaving the possibility that varying dosages or exposure time could produce divergent outcomes. In particular, the non-linear dose–response pattern between caffeine exposure and glucose tolerance needs further clarification. Finally, the experiment and follow-up period for offspring lasted PW16. All assessments were cross-sectional rather than longitudinal, and we could not determine whether the observed fetal outcomes in offspring, such as neurobehavior, metabolism, or circadian rhythm, will persist into adulthood or disappear with age. Future investigations should strive to mitigate these limitations by exploring a more comprehensive spectrum of exposure parameters, extending the follow-up period to clarify the permanence or reversibility of these effects and utilizing experimental models or prospective cohort studies.

## 5. Conclusions

In summary, this study demonstrates that prenatal caffeine exposure significantly influences the weight gain, behavioral patterns, glucose metabolism, and circadian regulation in offspring rats. These findings provide critical insights into the potential risks associated with maternal caffeine consumption during gestation. Future research should focus on elucidating the molecular and genetic mechanisms behind different effects, providing precise evidence for strict intake limits of caffeine during pregnancy, and offering new ideas and targets for the treatment of related fetal diseases.

## Data Availability

The original contributions presented in this study are included in the article. Further inquiries can be directed to the corresponding author.

## Supplementary Materials

The following supporting information can be downloaded at: https://www.mdpi.com/article/10.3390/toxics14100893/s1, Supplementary Table S1: The effect of gestational caffeine exposure on pregnancy outcomes.
