# Supplementary material for: Effects of Maternal Caffeine Exposure During Gestation on Neurobehavior, Glucose Metabolism and Circadian Rhythm in Offspring Rats
Source: Toxics. 2026 Oct 7;14(10):893. doi: 10.3390/toxics14100893 (PMC13709815; doi:10.3390/toxics14100893)
Supplement: Supplementary file 1 [file toxics-14-00893-s001.zip › Supplementary Table S1.pdf]

Supplementary Materials

Supplementary Table S1. The effect of gestational caffeine exposure on pregnancy outcomes.

Group	Gestation length(d)	Little size	Pups birth weight(g)
control	21.3±0.5	13.5±3.7	7.10±0.98
5mg/kg caf	21.7±0.5	14.4±4.7	7.60±0.97
20mg/kg caf	21.6±0.5	14.1±4.1	6.97±0.76
80mg/kg caf	21.9±0.4	9.8±5.0	6.77±0.56

N=10-12 pregnant rats per group. All data are expressed as Mean±SD. One-way ANOVA with Dunnett's post hoc test were performed.
